# Urinary excretion of EGF and MCP-1 in children with vesico-ureteral reflux

**DOI:** 10.1590/S1677-5538.IBJU.2015.0132

**Published:** 2017

**Authors:** Valentina Pastore, Fabio Bartoli

**Affiliations:** 1 Department of Medical and Surgical Sciences, University of Foggia, Foggia, Italy

**Keywords:** Urinary Tract, EGF Family of Proteins, Vesico-Ureteral Reflux

## Abstract

**Purpose:**

The aim of this study was to investigate the urinary concentration of epidermal growth factor (EGF) and monocyte chemotactic protein-1 (MCP-1) as reflux nephropathy (RN) biomarkers before and after endoscopic treatment of moderate to severe vesico-ureteral reflux (VUR).

**Materials and methods:**

A prospective study was carried out on 72 children with moderate to severe VUR. All patients underwent endoscopic treatment using Macroplastique® or Deflux®. Vesico-ureteral reflux resolution was tested by post-operative voiding cystourethrography after 3 months and 2 years. Follow-up urinary samples were collected at that time. Control samples were taken from healthy children with no clinical evidence of renal and bladder disease and no history of UTI.

**Results:**

In VUR patients, pre-operative urinary EGF levels had a down-regulation when compared to controls. Following successful VUR repair, urinary EGF levels of VUR children progressively increased only at long term follow-up but without returning to normal levels. Urinary MCP-1 levels were highly expressed in pre-operative samples and decreased markedly during early post-operative measurements. Urinary MCP-1 levels did not further decreased in late post-operative follow-up. In fact, these levels remained significantly higher when compared to controls.

**Conclusions:**

Urinary levels of EGF and MCP-1 may become useful markers for monitoring the response to surgical treatment in VUR patients. Although endoscopic VUR treatment is effective in reducing the inflammatory response, the persistence of significant abnormal levels of inflammatory cytokines (such as urinary MCP-1) at long term follow-up suggests that surgery alone may not completely treat the chronic renal inflammation evidenced in these children.

## INTRODUCTION

Vesicoureteral reflux (VUR) is the most common urological disease in children, affecting nearly 1% of the general pediatric population (1). Vesicoureteral reflux is found in 25-70% of children with urinary tract infections (UTIs) (2). These children are at risk of pyelonephritis or renal scarring, which may progress to reflux nephropathy (RN) and end-stage renal damage (3, 4). The main histopathological findings of RN are tubular atrophy with prominent mononuclear inflammatory cellular infiltrate and interstitial fibrosis (5). The main biomolecular mechanisms responsible for its progression still under investigation are genetic, pro-inflammatory and pro-apoptotic. The kidney is an important site for the production of epidermal growth factor (EGF) (6) which play a major role in renal growth modulation and turnover of tubular cells, glomerular hemodynamics, renal metabolism, tubular transport and eicosanoid synthesis (7, 8). Tissue EGF down-regulation has been reported in several chronic renal diseases (8-10), in obstructive (11) and in RN (12). Monocyte chemotactic protein-1 (MCP-1) is a powerful and specific chemotactic and activating factor for circulating monocytes (13). Infiltrating monocytes contribute to a decline in renal function following acute and chronic renal injury (14). Our group has recently shown an inverse relationship between EGF and MCP-1 (decreased EGF and increased MCP-1) tissue expression in obstructive nephropathy (11), with similar findings also reported recently in renal biopsies obtained from nephrectomies secondary to RN (12). Nevertheless, the role of EGF and MCP-1 as biomarkers of renal damage progression isn’t totally elucidated and has been the subject of widespread interest in recent years. The aim of the present study was to investigate the role of EGF and MCP-1 urinary levels as biomarkers of RN before and after endoscopic treatment for VUR at long term follow-up.

## MATERIALS AND METHODS

A prospective study was carried out from 2006 to 2012 on 72 children (44 boys and 28 girls) from 1 to 12 years of age (mean 4 + 2.7 years) with primary moderate to severe VUR. Vesico-ureteral reflux was graded according to the International Reflux Study Committee by voiding cystourethrography (VCUG) as moderate (grade III) or severe (grade IV-V). All patients had been started on antibiotic prophylaxis for 1 year before being referred for surgery, unless high grade VUR with recurrent UTIs resistant to prophylaxis coexisted. Informed consent was obtained for endoscopic treatment of VUR, collection of urinary samples and use of medical records for scientific purposes. All the children had been free of UTIs for at least 1 month and all inflammatory indexes (fever, white blood count, c-reactive protein) were normal at the time of treatment or sample collection. None of the children included had abnormal serum creatinine. All patients underwent endoscopic treatment with both Macroplastique® or Deflux®. Vesico-ureteral reflux resolution was tested by post-operative VCUG after 3 months and 2 years. Further follow-up studies included renal ultrasound (US) at 1, 3, 6 and 12 months. All patients continued antibiotic prophylaxis for at least 3 months after endoscopic injection. At US follow-up, 5 asymptomatic children developed transitory urinary obstruction, which was resolved spontaneously within one year in 4 cases. One patient required ureteric reimplantation due to persisting ureterovesical obstruction. Urinary samples were collected at the time of surgical procedure (pre-operative) and at 3 and 24 months after endoscopic treatment (post-operative). Control urinary samples were obtained from 15 children who were free of systemic, renal or inflammatory diseases at the time of urinary collection. Control cases were selected after having enrolled affected children to match them for age and sex. Clinical characteristics of VUR patients included in the study are reported in Table-1.

**Table 1:** Clinical characteristics of vesico-ureteral reflux (VUR) patients included in the study.

Clinical Characteristics (number of pts)	Yes	No
Recurrent UTI	52	20
Bilateral VUR	25	47
Age range 12- 36 months	45	27
**Severe VUR**	49	23
Split Renal Function < 40%	29	43

### Enzyme-linked immunosorbent assay (ELISA)

Urinary samples were collected, with patients properly hydrated (on free fluid intake), before and at 3 and 24 months after surgery. Once collected, urinary samples were centrifuged at 10.000rpm x 10 minutes, then stored in a deep freezer at -80°C until tested. Because urinary EGF concentration is significantly higher than that of MCP-1, urinary samples were diluted by 2 and 200 before measurement of urinary concentrations of MCP-1 and EGF respectively. Quantitative measurements were obtained by using a human MCP-1 and EGF ELISA commercial kit (Quantikinine; R&D, Abingdon, UK, and Biotrak; Amersham, UK, respectively), a multiple sandwich solid-phase enzyme immunoassay that used monoclonal antibody raised against human MCP-1 and EGF. ELISA sensitivity was 5pg/mL for MCP-1 and 8pg/mL for EGF. The enzymatic reaction was detected in an automatic microplate photometer (Titertek; Flow Labs, Helsinki, Finland), with MCP-1 and EGF concentrations of unknown samples determined by interpolation into standard curve developed with known amounts of recombinant human MCP-1 and EGF. Urinary MCP-1 and EGF levels were normalized to urinary creatinine excretion and expressed as pg/mg urinary creatinine, respectively. The urinary levels of both EGF and MCP-1 were not only expressed as the ratio of cytokine to urinary creatinine as specifically advised by R&D but also by calculating the EGF/MCP-1 ratio (from now on simply referred to as Ratio). The Ratio was calculated by separately dividing the mean and SD of EGF and MCP-1, after which each result was multiplied by 100. The final results express RATIO [mean]+[SD] in Arbitrary Unit (AU).

**Example:**

RATIO [mean]=EGF [mean] x 100 MCP-1 [mean]

RATIO [SD]=EGF [SD] x 100 MCP-1 [SD]

### Statistical analysis

Single extreme values of each group were excluded, with data expressed as mean + SD. Quantitative data were compared among groups by analysis of variance and paired t-test, as appropriate. Correlation coefficients were Pearson’s r values. Differences were considered significant when p <0.05. The study of patient subgroups (age, gender, laterality, grade, UTIs, scars) is not reported in this paper since the number of patients and samples were insufficient to guarantee accurate statistical analysis for these subgroups.

## RESULTS

All numerical results (mean + SD) of urinary levels of EGF, MCP-1 and Ratio are reported in Table-2.

**Table 2:** Pre-and post-operative urinary concentration of EGF, MCP-1 and Ratio in control and in vesico-ureteral reflux patients.

	EGF pre-op (pg/mL) Mean ± SD	EGF post-op 3 (pg/mL) Mean ± SD	EGF post-op 24 (pg/mL) Mean ± SD	MCP-1 pre-op (pg/mL) Mean ± SD	MCP-1 post-op 3 (pg/mL) Mean ± SD	MCP-1 post-op 24 (pg/mL) Mean ± SD	EGF/MCP-1 pre-op (AU) Mean ± SD	EGF/MCP-1 post-op 3 (AU) Mean ± SD	EGF/MCP-1 post-op 24 (AU) Mean ± SD
CTRL	790 ± 190			85 ± 57			750 ± 260		
VUR p= values	681 ± 277 ^p=0.044	489 ± 251 ^p=0.001; *p=0.012	587 ± 198 ^p=0.031; *p=0.037; °p=0.042	194 ± 128 ^ p=0.012	110 ± 86 ^p=0.047; *p=0.001	101 ± 62 ^p=0.043; *p=0.001; °p=n.s.	410 ± 150 ^p=0.001	552 ± 190 ^p=0.025; *p=0.003	600 ± 140 ^p=0.042; *p=0.0011; °p=n.s.

^-Statistical differences with control

*-Statistical differences between pre-operative and post-operative

°-Statistical differences between post-operative

**Figure 1:**
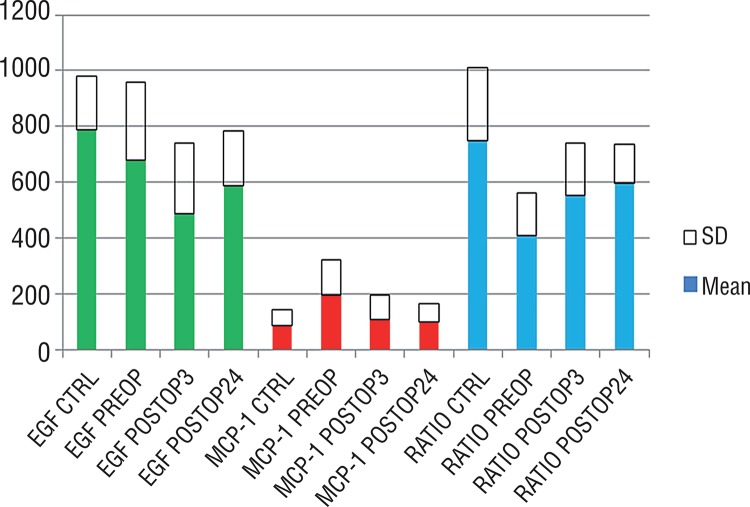
Pre-and post-operative urinary levels of EGF, MCP-1 and Ratio in control and in vesico-ureteral reflux patients.

Pre-operative urinary levels of EGF, MCP-1 and Ratio (Figure-1).

Pre-operative urinary levels of VUR patients vs control patients had a significant down-regulation of EGF (uEGF CTRL vs. uEGF VUR PRE-OP, p=0.044) and up-regulation of MCP-1 (uMCP-1 CTRL vs. uMCP-1 VUR PRE-OP, p=0.012). In addition, Ratio values had a marked decrease when compared to controls (Ratio CTRL vs. Ratio PRE-OP VUR, p=0.001). These findings demonstrate a strongly inflammatory response and decreased regenerative activity in patients with severe VUR prior to surgical treatment.

Post-operative urinary levels of EGF, MCP-1 and Ratio (Figure-1).

**A) EGF**

Unexpectedly, 3-month post-operative urinary EGF levels had a further decrease, which was statistically significant compared to pre-operative values (uEGF VUR PRE-OP vs. uEGF VUR POST-OP-3, p=0.012). However, urinary EGF concentration at 24 months follow-up demonstrated significant recovery when compared to that at 3 months follow-up (uEGF VUR POST-OP-3 vs. uEGF VUR POST-OP-24, p=0.042), although still lower than controls and PRE-OP (uEGF VUR POST-24 vs. uEGF CTRL, p=0.031; uEGF VUR POST-24 vs. uEGF VUR PRE-OP, p=0.037).

**B) MCP-1**

Post-operative urinary MCP levels-1 had a marked reduction when compared to pre-operative levels (uMCP-1 VUR PRE-OP vs. uMCP-1 VUR POST-OP-3, p=0.001; uMCP-1 VUR PRE-OP vs. uMCP-1 VUR POST-OP-24, p=0.001). However, despite the initial improvement in MCP-1 concentration, urinary concentrations measured at 24 months follow-up did not differ significantly from those measured at 3 months follow-up (uMCP-1 VUR POST-3 vs. uMCP-1 VUR POST-24, p=n.s). Furthermore, post-operative MCP-1 concentrations at 24 months follow-up remained significantly higher than in controls (uMCP-1 VUR CTRL vs. uMCP-1 VUR POST-24, p=0.043).

**C) Ratio**

Ratio measurements at 3 and 24 months follow-up had a clear and constant increase in values (Ratio VUR PRE-OP vs. Ratio VUR POST-OP-3, p=0.003; Ratio VUR PRE-OP vs. Ratio VUR POST-OP-24, p=0.0011) when compared to pre-operative urinary Ratio levels. Although the Ratio at 24 months was significantly reduced compared to CTRL, it continued to improve compared to that measured at 3 months follow-up (Ratio VUR POST-OP-3 vs. Ratio VUR POST-OP-24, p=0.04).

Figure-1 summarizes the mean and SD of pre-and post-operative urinary levels of EGF, MCP-1 and Ratio in CTRL and VUR patients.

## DISCUSSION

Post-operative results obtained from urinary samples of children with treated VUR clearly demonstrate decreased inflammatory response and a progressively better regenerative/inflammatory Ratio when compared to pre-operative results.

The histological hallmark of RN is chronic inflammation and tubular atrophy (5). The role of EGF as a renal growth factor is well documented in both animal and human studies. MCP-1, a potent and specific chemotactic agent for monocytes, has also been thoroughly investigated in experimental and clinical models of urinary tract obstruction (11). However, there is little data available regarding the changes of these cytokines in patients with RN and, to our knowledge, no reports about their changes before and after VUR endoscopic treatment. Although various studies have investigated tissue expression of EGF and MCP-1 on kidney remnants obtained from nephrectomies of children with severe RN, indicating a significant down-regulation of EGF and upregulation of MCP-1 (12), they failed to answer two questions: 1) are urinary levels of these cytokines useful in clinical practice in the monitoring of renal damage progression in RN? and 2) does surgical treatment of VUR improve RN outcome by decreasing inflammatory response? Our previous studies in children with urinary flow impairment secondary to ureteropelvic junction obstruction demonstrate that both EGF and MCP-1 are useful biomarkers for monitoring renal damage progression before and after surgical repair (14).

The aim of this study was to monitor these biomarkers in patients with VUR who needed endoscopic VUR treatment. In the past 20 years, endoscopic treatment of VUR has become the treatment of choice for all VUR grades, at our Institute as well (15).

We were concerned that the foreign body reaction which invariably takes place in these patients after bulking agent injection could have constituted a bias (16, 17). In fact, although foreign body reaction to bulking agents is important to stabilize the implant, for the purposes of this study it may have increased the urinary concentration of MCP-1. In order to minimize this possible bias, we set the first urinary sample collection at 3 months after endoscopic injection.

Indeed, in patients with RN we observed a pre-operative down-regulation of urinary concentration of EGF when compared to CTRL although, interestingly, even this finding worsened 3 months after VUR treatment. In contrast, urinary EGF levels returned to pre-operative levels at 24 months follow-up. No patient had post-operative EGF urinary concentration returned to normal levels. Urinary MCP-1 levels seem to follow a different pattern. In fact, they are highly expressed in preoperative samples and improved markedly during early post-operative measurement, after which they remain stable on late post-operative follow-up although still higher than those measured on CTRL samples. It is our opinion that MCP-1 increases mainly as a result of acute tubular damage secondary to VUR , while its prompt reduction following surgical VUR treatment demonstrates a positive anti-inflammatory action of VUR correction. However, children with treated VUR still have abnormal levels of MCP-1 at long term follow-up. These findings may suggest the presence of chronic renal or urinary inflammation persisting over time.

Furthermore, Ratio analysis provides a clearer view of changes occurring in these children, as this Ratio showed a marked decrease in pre-operative urinary samples and significant improvement in samples obtained at both early and late follow-up. However, post-operative Ratio levels continued to be significantly down-regulated when compared to CTRL, a result which lends further support to the opinion that some forms of poor tubular regeneration or persisting chronic inflammation still continue in these children.

Indeed, it has been demonstrated that in animal models a fibrotic process continues following treatment with progressive renal damage as a consequence of inflammatory response and renal apoptosis (18, 19). The end-point is progressive and chronic fibrosis which, in many cases, produces a hypoplastic kidney or chronic renal failure. The fibrotic response is characterized by thickening of the tubular basement membrane and widespread fiber accumulation in the tubule-interstitial compartment, defined as tubule-interstitial fibrosis (TIF) (20). The pathophysiology of TIF seems to be the response to a range of cellular stress, alteration of cellular death/proliferation and development of renal inflammation. Interleukins are secreted by injured tubular and urinary epithelium in infectious and non-infectious urinary tract conditions (21, 22). IL-6 seems to be associated with the presence of high grade VUR while IL-8 is linked to scar formation (23). Recent studies (24) point to a relationship between kidney injury molecule-1 (KIM-1), a trans-membrane protein and a marker of tubular damage, and severity of scar formation while urinary excretion of Beta 2-microglobin has been tested as a urinary marker of tubular damage and scar formation in VUR patients (25, 26). In addition, MCP-1 and macrophage colony stimulating factor (M-CSF) facilitate the homing of inflammatory cells to areas of tubular damage (27). The cellular death as a consequence of inflammatory response occurs at two levels: first, due to myeloperoxidase release by macrophages and neutrophils which catalyzes the production of toxic pro-oxidants such as hypochlorous and nitric acids; and second, due to tumor necrosis factor alpha (TNF-alpha) and Fas Ligand originating from those cells and latterly from the arrival of T-cells. These cytokines constitute the major receptor-triggered apoptotic signals in nephrons following urinary obstruction (28, 29), with a key role also played by transforming growth factor-beta 1 (TGF-β1), regarded as the pro-fibrotic factor “par excellence”.

A popular theory suggests that fibrosis occurs through a trans-differentiation process known as epithelial-to-mesenchimal trans-differentiation (EMT) and that TGF-β1 directly induces EMT (this phenomenon has been tested in cultured tubular epithelial cells) especially when EGF is added (30). Therefore, as we have demonstrated in these children, the persistence of abnormal levels of urinary MCP-1, may contribute to maintaining TIF and EMT. From a clinical point of view, our results confirm the need for some medical therapy able to further down-regulate the inflammatory response in the long term follow-up. We are still unable to suggest new drugs useful for this purpose because most of them are still under basic experimental steps. Steroids may be an option but their adverse effects are still a great problem. The main limit of this research is the small number of cases enrolled which has limited the statistical analysis of patient’s subgroups. To correct this, we are planning an international multicenter study to confirm the results and to further analyze the clinical significance of our preliminary data.

## CONCLUSIONS

In conclusion, we believe that, in the future, urinary levels of EGF and MCP-1 and above all their Ratio may become useful markers for monitoring response to surgical treatment in VUR patients. Additionally, a multicentric study is needed for better analysis of these urinary biomarkers in patient subgroups, particularly in relation to the presence of renal scars. Although endoscopic treatment of VUR is effective in significantly reducing the inflammatory response triggered by VUR, the persistence at long term follow-up of abnormal levels of inflammatory cytokines suggests that successful surgery alone may not completely treat the chronic renal inflammation evident in these children. The development of new, effective and minimally toxic medical therapies able to modulate the cascade of inflammatory, apoptotic and pro-fibrotic events occurring in VUR is the next challenge to be faced.
